# Development of Treatment Concepts for the Use of Botulinum Toxin A in Children with Cerebral Palsy

**DOI:** 10.3390/toxins2092258

**Published:** 2010-08-27

**Authors:** Richard Placzek, Dagmar Siebold, Julia F. Funk

**Affiliations:** 1 Department of Paediatric Orthopaedics, Centre for Musculoskeletal Surgery, Charité-Universitätsmedizin Berlin, Augustenburger Platz 1, 13353 Berlin, Germany; Email: julia.funk@charite.de; 2 Centre for physiotherapy and neurorehabilitation, Berlin, Germany; Email: dagmar.siebold@web.de

**Keywords:** botulinum toxin A, cerebral palsy, CP, multi-level treatment, key-muscle concept

## Abstract

The treatment of children with cerebral palsy with Botulinum toxin A injections is well established, safe and effective. However, a standardized injection strategy is still missing and the used dosage has escalated over the years. In the recent past, the recommended dosages in Europe were, however, reduced due to a better understanding of the relationship between dosage, severe side effects and the kind of anesthesia used. To combine safety and efficacy, the trend tends to a lower dosage, but combined with a more specific selection of injected muscles. The treatment of these key-muscles takes into account the best support for motor development to attain each individual motor milestone.

## 1. Introduction

### 1.1. Background

The American orthopedic surgeon A.L. Koman first described the use of botulinum toxin A for the treatment of cerebral palsy (CP) in 1993 [1]. Since then therapy with botulinum neurotoxin type A (BoNT-A) has been established as an important pillar of treatment within the therapeutic options for these patients. The therapy is regarded as safe and effective and, following adequate BoNT-A injection protocols, has been shown to lead to a reduction of surgical interventions [2]. It is currently considered to meet the criteria of evidence based medicine as a therapeutic option for treating spasticity in children and adults [3].

### 1.2. Rationale for BoNT-A Application

The clinical symptoms of cerebral palsy (CP) are caused by non-progressive damage to the central nervous system. This damage is permanent but, in particular during growth, its effects are not unchanging. In Europe, in approximately 90% of the affected children this damage leads to the clinical symptoms of spastic cerebral palsy, in about 6% to the dyskinetic form and in 4% to the ataxic form [4]. Direct consequences include, in addition to abnormal muscle tone, the loss of selective muscle control and disturbance of physiological balance mechanisms which ultimately lead to limitation of motor development. 

As secondary consequences of spasticity, particularly during growth, structural contractures can occur such as hip and knee flexor and adductor contractures and structurally fixed pes equinus. Furthermore, alterations such as coxa antetorta, tibial torsion and pes planovalgus, summarized by the term “lever arm diseases”, as well as spasticity-related hip luxation and subluxation are common resultant bony deformities.

Based on its mechanism of action as an inhibitor of the release of acetylcholine, the use of BoNT-A is targeted at the reduction of spasticity. Contractures that are already structurally fixed can hardly be treated. Therefore, when deciding whether treatment with BoNT-A is indicated, it must be carefully differentiated during clinical examination between dynamic (spasticity) and structural components (contracture) of movement limitation. 

**Figure 1:**
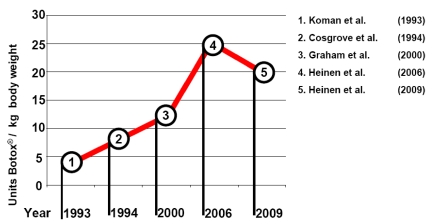
Reported/recommended total dose of Botox^®^ for the treatment of infantile cerebral palsy by: (1) Koman *et al.* [1]; (2) Cosgrove *et al.* [11]; (3) Graham *et al.* [12]; (4) Heinen *et al.* [13]; (5) Heinen *et al.* [14].

In children with CP, the motor development involved in verticalisation and locomotion usually takes place before the age of seven years [5]. By the age of five about 60% of all children with total body involvement (bilateral spastic cerebral palsy of upper and lower extremities) reach the ability to walk, another 10% reach it by the age of ten [6]. Based on this data there is wide agreement that in order to promote functional gait early, BoNT-A application should be performed, *i.e.*, beginning before the age of six years [7]. Although currently available clinical studies have, at best, yielded clear evidence for the successful treatment of spastic pes equinus and none at all for regimens including total lower and upper extremity treatment [8], the integration of BoNT-A therapy into a comprehensive treatment concept is generally required. In clinical routine, BoNT-A can be frequently integrated into existing treatment regimens when indicated according to current appropriate integrative patient and therapy management or multimodal therapy. Modifications of orthoses, physiotherapy, occupational therapy, medication, *etc.* may be necessary. Occupational therapy and physiotherapy play a special role since there will be improved therapeutic opportunities during the period from the effect of BoNT-A. Due to the reduction of spasticity, a “therapeutic window” for treatment in general, in particular the promotion of development and the prevention of contractures, is opened for this period. When treating children their social settings (family, school/care institution, hobbies, friends, *etc.*) should be particularly taken into account. Hence, *in praxi* the BoNT-A therapist must maintain close communication with associated disciplines as well as flexibility and pragmatism. However, to date there is no uniform treatment strategy and the doses used have significantly varied over the years [9]. The increase in the total doses of Botox^®^ used, measured in units/kg body weight (BW), is shown in Figure 1. These total doses are not evidence based, but are based on “expert opinion”. Randomized, double-blind, placebo-controlled, dose-ranging studies such as the one from Baker *et al.* 2002 [10] are the rather rare exceptions concerning dose ranging studies with BoNT-A.

In the recent past, the recommended dosage in Europe was reduced due to a better understanding of the relationship between dosage, severe side effects and the kind of anesthesia used.

Currently, within the clinical management of CP, the use of botulinum toxin is recommended to improve function and to support motor development [15].

### 1.3. Safety

Severely affected CP patients (GMFCS Level V; [16]) are particularly prone to side effects. Organ systems of the patients that function within a compensatory status can decompensate as a result of slight weakening, which might cause undesirable effects such as aspiration, pneumonia and asphyxia [17]. Considering data currently available, and the current discussion of serious side effects as a consequence of overdose [18,19,20], the manufacturers’ specifications (for Dysport^®^) or conservative dosage recommendations (for Botox^®^; [12]) should be complied with, in particular for severely affected children. It has been suggested that sedation and anesthesia are also risk factors, in addition to the total dose [21]. 

## 2. Previous Development

Based on the results of instrumental gait analysis and differential clinical examination procedures, surgical treatment of CP has fundamentally changed in the past two decades and the necessity of early multi-level treatment is now taken into account. The concept being that, as far as possible, all muscle groups involved should be surgically treated in the same session [22]. Despite this, according to more recent publications, it must be borne in mind that the manifestation of spasticity significantly changes during the growth period and that at around the age of 12 years, a reduction in spasticity can be expected [23]. The same authors also state that early selective treatment significantly reduces or even avoids the necessity for later multi-level treatment [24].

Analogously to multi-level surgical treatment, the spectrum of botulinum toxin therapy has been significantly widened with respect to the number of muscles or muscle groups treated. 

Over time the original single level injections for the treatment of pes equinus have been replaced by multi-level injections (e.g., M. gastrocnemius, medial knee flexors, adductors and M. psoas).

### High-Dose Multi-Level BoNT-A Therapy

The ultimate aim of this therapy, for which there is no generally agreed procedure, is an optimum alignment of the joints of the lower extremities to the pelvis by a treatment series analogous to surgical treatment.

This implies that if the distal muscles are exclusively involved then BoNT-A is only injected in these muscles. In children with more severe spasticity on the other hand each target muscle on each level should be injected. Through application of this regimen, the total dose for multi-level treatments of 2 to 6 muscle groups may be increased from 6 to 28 units per kg BW [25].

An essential part of multi-level therapy is the multimodal treatment approach. Plaster casts, physiotherapy and orthoses are particularly important. Treatment by plaster casts and functional and positioning orthoses in addition to physiotherapy is designed to improve longitudinal muscle growth and, thus, results in functional improvement. Within the scope of an integrated concept these options are combined to measurably increase the intensity and duration of the BoNT-A effect. 

To date, neither a standardization of this comprehensive multi-level concept exists nor may any clear conclusion be drawn from the presently available study data [8]. There are no studies concerning the ideal time to apply redression casts with regard to BoNT-A injection [26]. To avoid excessive inactivity atrophy of the non-spastic muscles, it is recommended that plaster casts be applied for no more than two weeks [7].

## 3. Key-Muscle Treatment

This concept, which was introduced by the author in 1998 and has been used and developed further since [27,28], comprises an advanced multimodal therapeutic approach as a development of the high-dose multi-level concept. The therapy has been specified to better attend to the special qualities and complexity of CP. If children with CP reach a higher level of motor development, they benefit from what they have learned for a period of time measurably longer than the pharmacological effect of botulinum toxin persists.

Generally speaking, children with CP have a risk of acquiring fixed contractures until the end of longitudinal growth. Hence, BoNT-A must stay available as a treatment option to these children for many years. Inadequate development of selective body control and balance due to abnormal muscle tone being the primary problems of spasticity as well as the secondary development of structural contractures and bone deformities (lever arm diseases), must be considered when planning treatment. Due to the increasing structural contractures and bone deformities, with the decreasing effect of BoNT-A in older children and adolescents for most children with cerebral palsy, surgery will be a preferred option at some point in time. Despite that, a continuation of therapy with BoNT-A even after surgery seems reasonable to avoid recurrence of contractures. 

Therefore, the characteristics of therapy have to be: long-term applicability, sustainability, individual and flexible planning. This leads to good acceptance by parents and therapists and also takes motor development into account. A basic requirement for long-term treatment is the avoidance of secondary non-response and the formation of antibodies. Thus, within the context of the key-muscle concept, multi-level injections are performed that strictly comply with well-proven dosage recommendations (see below).

For children with CP, motor development and motor learning are prolonged and difficult and there is a risk of long-term secondary changes. Therefore, the target of the key-muscle concept is to retain the treatment option with botulinum toxin for as long as possible, ideally during the entire development. In practice, the integration into a comprehensive interdisclipinary multimodal concept, including intensive physiotherapy, occupational therapy and orthoses, is essential. The key-muscle concept presented here takes these elements into account and is characterized by the following four points:

1. Treatment goal: reaching the next motor milestone, the next stage of physiological motor development;
2. Selection of the key-muscles;
3. Early commencement of treatment;
4. Long-term treatment option.

### 3.1. Treatment Goal: Reaching the Next Motor Milestone

The primary aim is to attain the next motor milestone with the prospective goal of verticalization and achieving the best possible locomotion. If a child reaches the ability to walk, the intention is to maintain, improve and optimize mobility. In case of stagnation on a lower motor level, the intention is to maintain, improve and optimize motor function on this level. 

**Figure 2:**
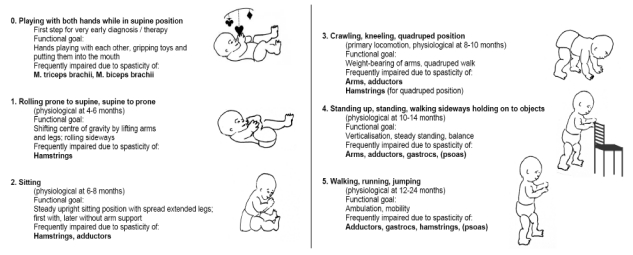
Classification of levels of motor development (schematic) as applied in the key-muscle concept.

The motor milestones have been defined according to Petö [29], the Gross Motor Function Measure (GMFM; [30]) and the WHO classification [31] as shown in Figure 2.

### 3.2. Selection of the Key-Muscles

Key-muscles are those muscles that, due to their spasticity, prevent attainment of the next motor milestone. Additionally, with respect to the key-muscle concept, muscles at acute risk for contracture or even muscles with initial contracture are injected.

Spastic muscles do not have to be injected as long as the elevated tone does not impair function, there is no acute risk for developing contractures or, if the tone permits, compensatory mechanisms.

### 3.3. Early Commencement of Treatment

The rationale for starting treatment as early as possible is based on the finding that already in the first year of motor development voluntary movements are organized around behavioral objectives. Children try out a combination of maneuvers to achieve a goal, thus learning new and more rapid movements and their coordination. To attain stable function, repetitive performance is necessary. In children with CP there may be an incorrect combination of movements. As a consequence, multiple repetitions lead to non-physiological learning, which results in non-physiological motor development or to the neglect of the affected extremity. It has been suggested that non-physiological movements lead to persisting effects in a developing brain [32]. During physiological development of the brain there is a progressive reduction of the cortical area activating motor function and hence an increase in the selective and specific control of movement [33]. After about one to two weeks, a brain lesion leads to hyperactivity of secondary ipsi- and contralateral motor areas which are, however, less numerous and less excitatory than the primary motor area [34]. A precondition for long-term improvement is the replacement of this secondary hyperactivity by activation of the primary stimulus, which is impossible in severe cases [35,36,37]. Furthermore, after several months deterioration, it is assumed to be due to inhibition of the affected motor area by the contralateral area [38]. Therefore, early improvement of the impaired motor function is plausible, and with an early commencement of treatment a great potential for development still exists. Non-physiological movement patterns are not learned in the first place, “dead ends” of motor development may be avoided. The importance of the first two years of life has to be taken into account—in physiological development all five motor milestones are reached during this period of time. The first years of life are also important concerning spasticity-related hip lateralization and dislocation [39]. Sometimes not reaching the ability to play with the hands in supine position is noticed very early (highlighted in grey in Figure 2). In such cases, there is good experience with early reduction of the tone of the extensors and flexors of the forearm. The risk profile for the recommended doses for children under two years of age is not different from that of older children [40].

An early treatment of muscles prone to shortening helps to prevent fixed contractures so that the number and extent of later surgical interventions can be reduced [41]. Sufficient physiotherapeutic support is essential for the success of early treatment according to the key-muscle concept. Therefore, an out-patient neurological rehabilitation program known as Berlin Model [42] has been provided to our patients in recent years.

### 3.4. Long-Term Treatment Option

A basic requirement for the long-term treatment option is to avoid secondary non-response and formation of antibodies. Hence, within the context of the key-muscle concept multi-level injections are performed with strict adherence to proven dosage recommendations (for first injection of Dysport^®^: 20 units/kg BW, subsequent injections: 10–30 units/kg BW, total dose: max. 1000 units Dysport^®^ (European Marketing Authorization); for Botox^®^: up to 12 units/kg BW, total dose: max. 300 units; [12]).

## 4. Two Case Reports Exemplifying BoNT-A Therapy According to the Key-Muscle-Concept

**Figure 3:**
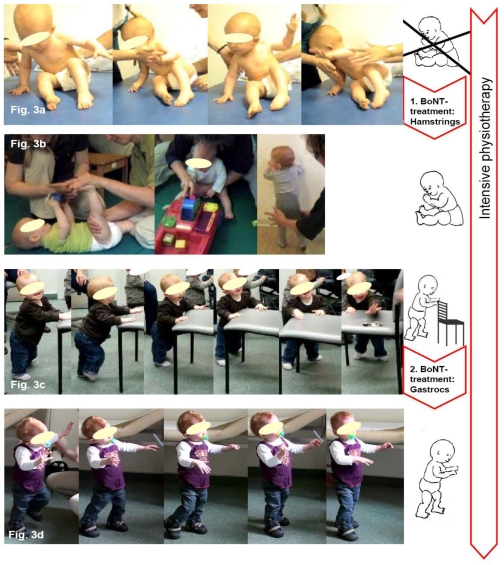
Case example of early BoNT-A therapy: 11 months old girl with bilateral spastic cerebral palsy (diplegia). (**a**) At first presentation, spasticity in the entire lower extremity was seen. The clinical examination revealed pronounced catch of the hip flexors, adductors, hamstrings and a spastic pes equinus on both sides (Ashworth 2–4). The targeted motor milestone—the ability to sit—could not be achieved, due to spastic shortening of the hamstring muscles. Meeting the requirements of the key-muscle concept the hamstrings were injected with 70 units Dysport^®^ each (total dose of 140 units at 7 kg BW, 20 units/kg BW respectively). (**b**) After 4 weeks of intensive physiotherapy according to the Berlin model the ability to sit was attained. (**c**) Sixteen weeks after BoNT-A injection, standing and walking sideways holding on to objects was possible. To improve the ability to stand and walk both Mm. gastrocnemii were injected to assist plantigrade placement of the feet. (**d**) After another 16 weeks the patient could walk for a few meters without aid. The clinical examination still showed reduced spasticity when compared to the previous examinations, hence no further BoNT-A injections were performed. Intensive physiotherapy was continued and it was agreed that the patient would be presented again after another 8 weeks.

**Figure 4:**
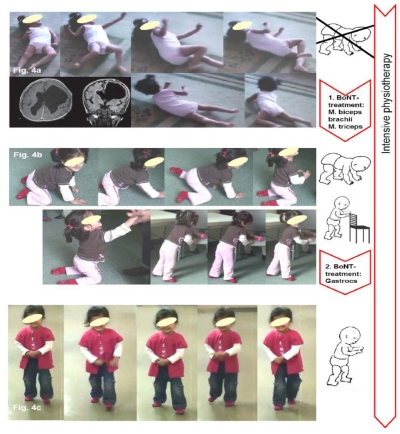
Case example of specific selection of the key-muscles: 3 year old girl with right-sided unilateral spastic cerebral palsy. (**a**) At first presentation, crawling as a motor milestone was impossible. The girl was able to roll from supine to prone position and *vice versa* with her right arm held in flexion due to spasticity. Hence, rolling was her form of moving about. No speech (not even single words) had developed. The neuropediatric diagnosis was spastic hemiplegia with global retardation. Despite a multi-level BoNT‑A injection, elsewhere performed, in the lower extremity, and physiotherapy on a regular basis, no improvement of motor development was achieved. Two sections of the cranial MRI are shown in the bottom left. The clinical examination revealed pronounced catch of the flexors, as well as the extensors of the right upper arm (Ashworth 3). Therefore, BoNT-A was injected in those muscles (30 units of Dysport^®^ each) and intensified physiotherapy according to the Berlin model was started. To reach the motor milestone, crawling extension and weight-bearing of both arms is necessary and a treatment goal. (**b**) 6 months after injection and intensive physiotherapy the motor milestone crawling was achieved. Standing and walking sideways holding on to objects is possible with a spastic equinus and a compensatory recurvation of the knee on the right side, as well as trunk inclination for balance and stability. To further improve the ability to stand and walk, the right M. biceps brachii and the right M. gastrocnemius were injected with 30 and 100 units, respectively, of Dysport^®^ and physiotherapy continued. A planned visit 4 months later is omitted. (**c**) At the next presentation one year later the patient is able to walk without support. She had attended physiotherapy on a regular basis. The girl is now able to speak simple sentences.

Two case reports BoNT-A Therapy according to the Key-Muscle-Concept are detailed in Figure 3 and Figure 4. Figure 3 details the case of an 11 month-old girl with bilateral spastic cerebral palsy (diplegia) and Figure 4 shows a three year-old girl with right-sided unilateral spastic cerebral palsy.

## 5. Conclusions for Practice

Treatment of CP with Botulinum toxin is safe if the recommendations and guidelines for dosage are taken into account. The intermediate to long-term treatment results depend mainly on the correct indication and integration into a multimodal management concept, which has to include all other necessary treatment options such as orthoses, physiotherapy, occupational therapy, medication, *etc.* It is not a case of “either …or”, but “…as well as…”. To establish whether treatment with BoNT-A is indicated, a detailed clinical analysis with differentiation of dynamic and structural components is essential and the definition of a treatment goal is absolutely necessary. To take into account the patients’ changes during growth, injection therapy must be adaptable, thus, it should be performed flexibly and individually. To avoid antibody formation during long-term therapy, re-injection intervals should be at least three months. 

## 6. Keystones of the Own Procedure in Accordance with the Key-Muscle Concept

### 6.1. Philosophy

Long-term multi-level injection treatment with strict adherence to the dosage recommendations of the company Ipsen (European marketing authorizations) and the work group around Graham *et al.* [12]: 20 (initial dose) up to 30 units Dysport^®^/kg/BW and up to 12 units Botox^®^/kg/BW.

### 6.2. Treatment Objective

Individual definition of management regimen for each patient, with optimum support of motor development, taking into account the developmental level. Early commencement of treatment as soon as motor development, or attaining a level of motor development corresponding to age, seem to be impaired due to spasticity.

### 6.3. Technique of Injection

At the lower extremity one skin puncture and, if necessary (greater quantity), injection in a fan-shaped pattern. Injections on an out-patient basis, if possible, so that the social settings of the children are interrupted as little as possible. Meticulous clinical orthopaedic examination to define the muscles to be injected (spastic muscles are easy to palpate without anesthesia; injection control of deep muscles or of muscles difficult to palpate using ultrasound). Best possible accuracy of injection with the premise that the child be affected as little as possible. General anesthesia only if absolutely necessary (e.g., for injecting the M. psoas). Minimum injection interval of at least three months.

### 6.4. Injection Solution

Use of a standard solution (500 units Dysport^®^ in 5 mL NaCl 0.9%, *i.e.*, 100 units Dysport^®^ per 1 mL or 100 units Botox^®^ in 2.5 mL NaCl 0.9%, *i.e.*, 40 units Botox^®^ per 1 mL).

### 6.5. Multimodal Therapy

Combination with physiotherapy, occupational therapy, orthoses, *etc.* (not **either** BoNT-A **or** other procedures, but BoNT-A **and** other procedures). No use of redression casts in favor of corrective splinting and intensification of physiotherapy.

### 6.6. Documentation

Written documentation of findings and video documentation during each clinical examination.
